# Suppression of PTPN6 exacerbates aluminum oxide nanoparticle-induced COPD-like lesions in mice through activation of STAT pathway

**DOI:** 10.1186/s12989-017-0234-0

**Published:** 2017-12-12

**Authors:** Xiaobo Li, Hongbao Yang, Shenshen Wu, Qingtao Meng, Hao Sun, Runze Lu, Jian Cui, Yuxin Zheng, Wen Chen, Rong Zhang, Michael Aschner, Rui Chen

**Affiliations:** 1 Key Laboratory of Environmental Medicine Engineering, Ministry of Education, School of Public Health, Southeast University, Dingjiaqiao 87, Nanjing, 210009 China; 2 Center for New Drug Safety Evaluation and Research, China Pharmaceutical University, Nanjing, China; 3 School of Public Health, Qingdao University, Qingdao, 266021 China; 4 Guangzhou Key Laboratory of Environmental Pollution and Health Risk Assessment, Department of Toxicology, School of Public Health, Sun Yat-sen University, Guangzhou, 510080 China; 5 Department of Toxicology, School of Public Health, Hebei Medical University, Shijiazhuang, 050017 China; 6 Department of Molecular Pharmacology, Albert Einstein College of Medicine, Bronx, NY 10461 USA; 7 Institute for Chemical Carcinogenesis, Guangzhou Medical University, Guangzhou, 511436 China

**Keywords:** Aluminum oxide nanoparticles; PTPN6, Experimental COPD, Inflammation

## Abstract

**Background:**

Inhaled nanoparticles can deposit in the deep lung where they interact with pulmonary cells. Despite numerous studies on pulmonary nanotoxicity, detailed molecular mechanisms of specific nanomaterial-induced lung injury have yet to be identified.

**Results:**

Using whole-body dynamic inhalation model, we studied the interactions between aluminum oxide nanoparticles (Al_2_O_3_ NPs) and the pulmonary system in vivo. We found that seven-day-exposure to Al_2_O_3_ NPs resulted in emphysema and small airway remodeling in murine lungs, accompanied by enhanced inflammation and apoptosis. Al_2_O_3_ NPs exposure led to suppression of PTPN6 and phosphorylation of STAT3, culminating in increased expression of the apoptotic marker PDCD4. Rescue of PTPN6 expression or application of a STAT3 inhibitor, effectively protected murine lungs from inflammation and apoptosis, as well as, in part, from the induction of chronic obstructive pulmonary disease (COPD)-like effects.

**Conclusion:**

In summary, our studies show that inhibition of PTPN6 plays a critical role in Al_2_O_3_ NPs-induced COPD-like lesions.

**Electronic supplementary material:**

The online version of this article (10.1186/s12989-017-0234-0) contains supplementary material, which is available to authorized users.

## Background

Airborne nanoparticle-induced pulmonary toxicity has been widely reported. This toxicity is largely dependent on the chemical and physical characteristics of specific nanoparticles. Pulmonary inflammation plays a critical initial event in metal oxide nanoparticle-induced respiratory disorders [1–3].

Aluminum oxide nanoparticles (Al_2_O_3_ NPs) have been widely used in the chemical, industrial and medical fields [4]. Al_2_O_3_ NPs represent one of the most abundantly produced nanosized particles, accounting for approximately 20% of the 2005 world market of nanoparticles [5]. The limit values for inhaled aluminum (Al) compounds remain relatively high. For instance, the United States Occupational Safety and Health Administration (OSHA) has set a legal limit (PEL) of 15 mg/m^3^ (total dust) and 5 mg/m^3^ (respirable fraction) for alumina in dusts averaging over an 8 h work day [6]. In total alumina dusts, nano-scaled particles exhibit a slower precipitation in air than the bulk, therefore increasing exposure duration in humans [7]. It is noteworthy that asthma has been recognized as one of the most prevalent pulmonary diseases in the aluminum exposure occupational setting [4]. In addition, insoluble Al compounds appear to be biopersistent in lung tissues. As demonstrated in rat intratracheal instillation model, only ~9% of Al_2_O_3_ was cleared from the lungs during a 19-week period following weekly instillation for 20 weeks [8]. Considering the high discharge levels during the manufacturing process [7], concern over the biosafety of Al_2_O_3_ NPs is warranted.

In term of toxic mechanisms, exposure to Al_2_O_3_ NPs has been shown to cause mitochondrial dysfunction in a dose-dependent manner in human fetal lung fibroblasts (HFL1) [9] and human bronchial epithelia HBE [10]. Other studies have shown the number of macrophages in bronchoalveolar lavage fluid (BALF) of Al_2_O_3_ nanowhisker-exposed mice to be two-fold higher than in control mice [11]. A series of studies by Pauluhn have further suggested pulmonary inflammation induced by high dose (28 mg/m^3^) Al exyhydroxides nanoparticles exposure [12, 13]. Taken together, these studies suggest a possible toxic mechanism involving oxidative stress and inflammation; however, detailed molecular mechanisms by which Al_2_O_3_ NPs interact with the pulmonary system have yet to be defined.

Our previous in vitro study suggested that suppression of protein tyrosine phosphatase, non-receptor type 6 (PTPN6) expression level in A549 cells plays a key role in Al_2_O_3_ NPs-induced cellular toxicity [14]. PTPN6 has been shown to be a critical negative regulator of intracellular signaling; it is predominantly expressed in hematopoietic cells and epithelia [15, 16]. Suppression of PTPN6 augments oxidative stress and exacerbates chronic inflammatory airway diseases [17]. Activation of the STAT3 pathway is involved in development of pulmonary inflammatory disease [18, 19], and PTPN6 has been recognized in multiple cell lines as a negative regulator of STAT3 [20–22]. In addition, up-regulation of PTPN6 has been shown to inhibit phosphor-STAT3 and mitigate activation of STAT3 in A549 cells [23]. Accordingly, we hypothesized that aberrant expression of PTPN6 might be involved in pulmonary disorders induced by Al_2_O_3_ NPs in vivo. To address this hypothesis, mice were exposed to Al_2_O_3_ NPs by dynamic inhalation to observe the alterations in lung function and pathology. We subsequently delineated the function of PTPN6 in Al_2_O_3_ NPs-related pulmonary disorders by integrating cellular assays with experimental mouse models. Notably, we show that rescue of PTPN6 expression levels significantly alleviates Al_2_O_3_ NPs-induced pulmonary inflammation as well as COPD-like lesions in murine lung tissues.

## Results

### Al_2_O_3_ NPs exposure increases levels of inflammatory mediators in mouse lung tissues

**Fig. 1:**
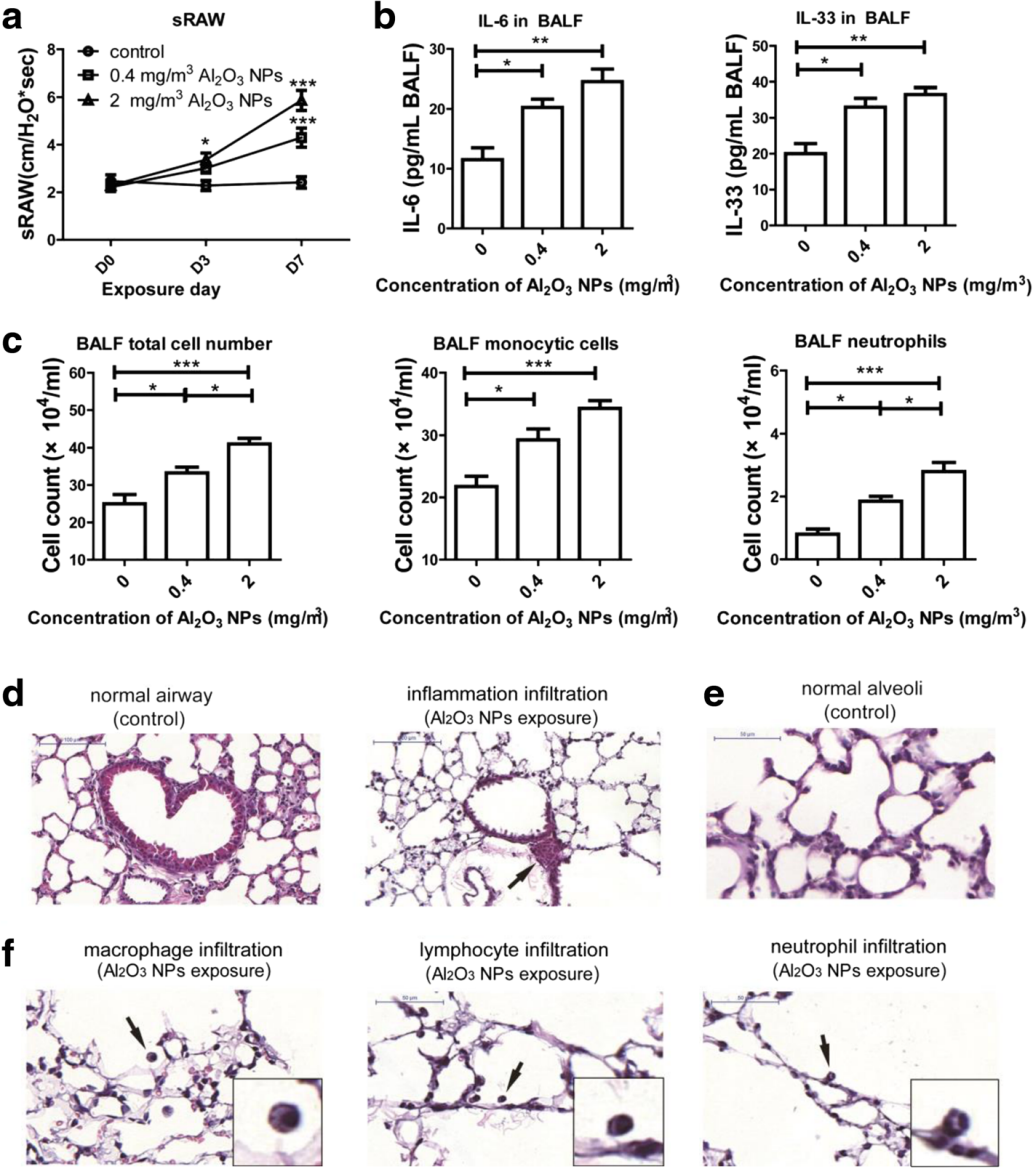
Pulmonary inflammation induced by Al_2_O_3_ NPs exposure. **a** sRAW was significantly increased in Al_2_O_3_ NPs-exposed mice (*n* = 10, ^*^ *P* < 0.05, compared with controls; ^***^ *P* < 0.001, compared with control on the same day). **b** Inflammatory mediators were significantly increased in Al_2_O_3_ NPs-exposed BALF of mice (*n* = 4, ^*^ *P* < 0.05, ^**^ *P* < 0.01). **c** The total cell number, monocytic cells number and neutrophils number in BALF of Al_2_O_3_ NPs-exposed mice were significantly increased compared with FRA control (*n* = 4, ^*^ *P* < 0.05, ^***^ *P* < 0.001). **d** Representative images of normal airway and airway surrounded by inflammatory cells (showed by arrow). **e** Representative images of distant alveoli (FRA control) **f** Representative images of macrophages, lymphocytes and neutrophil infiltration in alveolar area (Al_2_O_3_ NPs exposure) (shown by arrow). The images in the right bottom of each panel are magnified of the cell in original images highlighted with black arrows

Specific airway resistance (sRAW) (an index used to identify acute bronchial response [24]) was measured in conscious mice one day before Al_2_O_3_ NPs-exposure and on exposure days 3 and 7. The sRAW in 2 mg/m^3^ Al_2_O_3_ NPs-treated mice was significantly increased on day 3 and day 7 of exposure. In mice exposed to 0.4 mg/m^3^ Al_2_O_3_ NPs, a significant increase in sRAW was noted on exposure day 7 when compared with FRA control (Fig. 1a). Based on the findings that low levels of Al_2_O_3_ NPs exposure caused phenotype alteration on the 7th day of exposure, all the following experiments were carried out for 7 days.

The aluminum (Al) burdens in lungs of mice were 411 ± 68, 1238 ± 110 and 2951 ± 234 ng/g for the control (filtered-room air, FRA), 0.4 and 2 mg/m^3^ Al_2_O_3_ NPs-exposed groups, respectively. The 2 mg/m^3^ Al_2_O_3_ NPs-exposed groups had a 3-fold greater Al burden in the lungs compared to those exposed to 0.4 mg/m^3^ Al_2_O_3_ NPs. This may be related to greater damage to the lung in response to the higher dose, reflecting altered aggregation and deposition patterns.

**Fig. 2:**
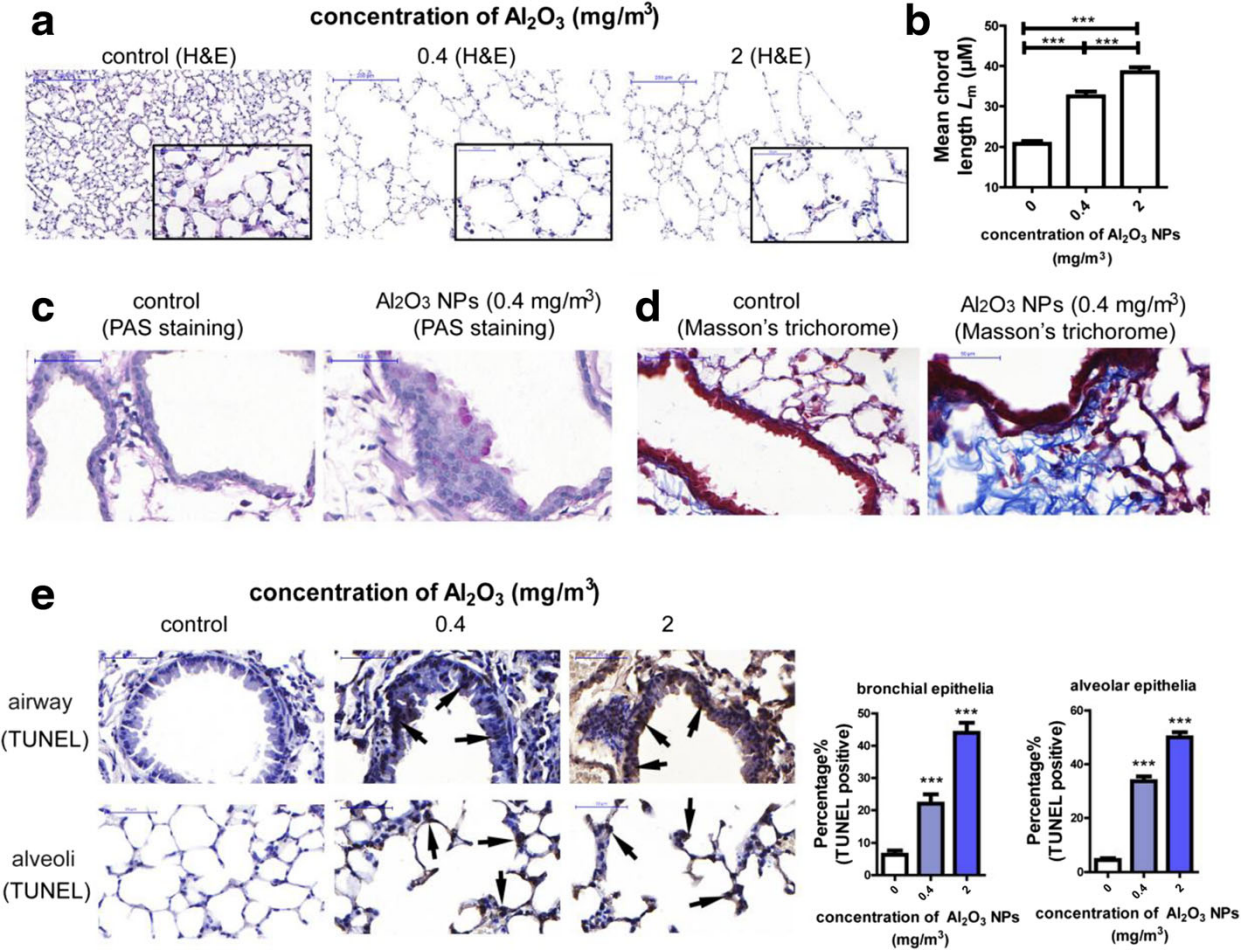
Exposure to Al_2_O_3_ NPs led to experimental COPD in a murine model. **a** Representative images of normal alveolar area and emphysema. The images on the right bottom of each panel are magnified (400) of area from the original one. **b** Lm was significantly increased in Al_2_O_3_ NPs-exposed mouse lungs (*n* = 36, ^***^ *P* < 0.001). **c** Representative images of PAS staining, the PAS^+^ cells suggested hypersecretion of airway epithelial cells (shown by arrows). **d** Representative images of Masson’s Trichorome staining. Deposit of collagen around airway was stained purple. **e** Representative images of TUNEL staining and percentage of TUNEL^+^ cells. The TUNEL^+^ cells were shown by arrows. (*n* = 30, ^***^ *P* < 0.001, compared with control group)

Compared to FRA exposed mice, Al_2_O_3_ NPs-exposed mice had higher concentrations of bronchoalveolar lavage fluid (BALF) interleukin (IL)-6 (a cytokine associated with decline in lung function [25]) and BALF IL-33 (a cytokine induces chronic airway inflammation [26, 27]) (Fig. 1b). Next, we assayed the total number of cells, number of monocytic cells and neutrophils in BALF. Compared with controls, the percentages of increased levels of IL-6 were 76% and 113%; increased IL-33 levels were 65% and 72.5; increased total cell numbers were 33% and 64%; increased monocytic cells were 34% and 57%, increased numbers of neutrophils were 130% and 250% in the 0.4 and 2 mg/m^3^ Al_2_O_3_ NPs exposed murine lungs, respectively. All of the above airway inflammatory indices were elevated in an Al_2_O_3_ dose-dependent manner when compared to FRA controls (Fig. 1c). Figure 1 d to g depict representative images of inflammatory infiltration in murine lung tissues, noting infiltration of inflammatory cells around small airways (Fig. 2d) (shown by arrow). Figure 1e showed the representative image of alveolar area of control murine lung. Increased infiltrating alveolar neutrophil, lymphocytes and macrophages (Fig. 1f) in response to Al_2_O_3_ NPs were also observed (shown by arrows).

### Al_2_O_3_ NPs exposure induce apoptosis and experimental COPD in mice

The pathological alterations in murine lung tissues were examined by hematoxylin and eosin (H&E) staining. We noted emphysema in the distant alveolar area, characterized by enlarged airspace and increased mean chord lengths (Lm) (established indices of experimental COPD [28]) in lung tissues after Al_2_O_3_ NPs exposures compared to FRA control (Fig. 2a and b). The other vital characteristic of experimental COPD, small-airway remodeling, was assayed by Periodic Acid-Schiff (PAS) [27] and Masson’s Trichrome staining [29]. Increased mucin glycoprotein secretion was detected by Periodic Acid-Schiff (PAS) staining, and mucus hypersecretion was observed in airway epithelial cells of Al_2_O_3_ NPs-exposed mice (Fig. 2c). Masson’s Trichrome staining was used to evaluate the deposition of collagen around small airway, a hallmark of airway remodeling. As shown in fig. 2d, the enhanced collagen deposition was enhanced in the lungs of Al_2_O_3_ NPs-exposed mice when compared with FRA controls. Exposure to ZnO NPs has been reported to lead to emphysema [30]; therefore, ZnO NPs were used as a positive control. It was noted that the major pathologic alterations in murine lungs induced by ZnO NPs occurred in the alveolar areas, characterized by thickened alveolar walls and emphysema (Additional file 1: Figure S1a). However, remodeling of airways has not been observed (data not shown). These results suggest that different metal oxide NPs exposure leads to varied pathologic alterations in the lungs.

Increased apoptosis of alveolar epithelial and endothelial cells in the lung is a vital upstream event in the pathogenesis of COPD, especially in the development of emphysema [31, 32]. Here, we detected apoptosis in the lungs of mice with the TUNEL assay. As shown in Fig. 2e, the percentage of apoptotic cells was significantly increased in a dose-dependent manner in airway epithelia and alveolar epithelia in the lungs of Al_2_O_3_ NPs-exposed mice compared with FRA control. Our observation strongly suggests that Al_2_O_3_ NPs exposure leads to inflammation and experimental COPD pathology in murine lungs.

### Suppression of PTPN6 resulted in overexpression of apoptosis related gene PDCD4

**Fig. 3:**
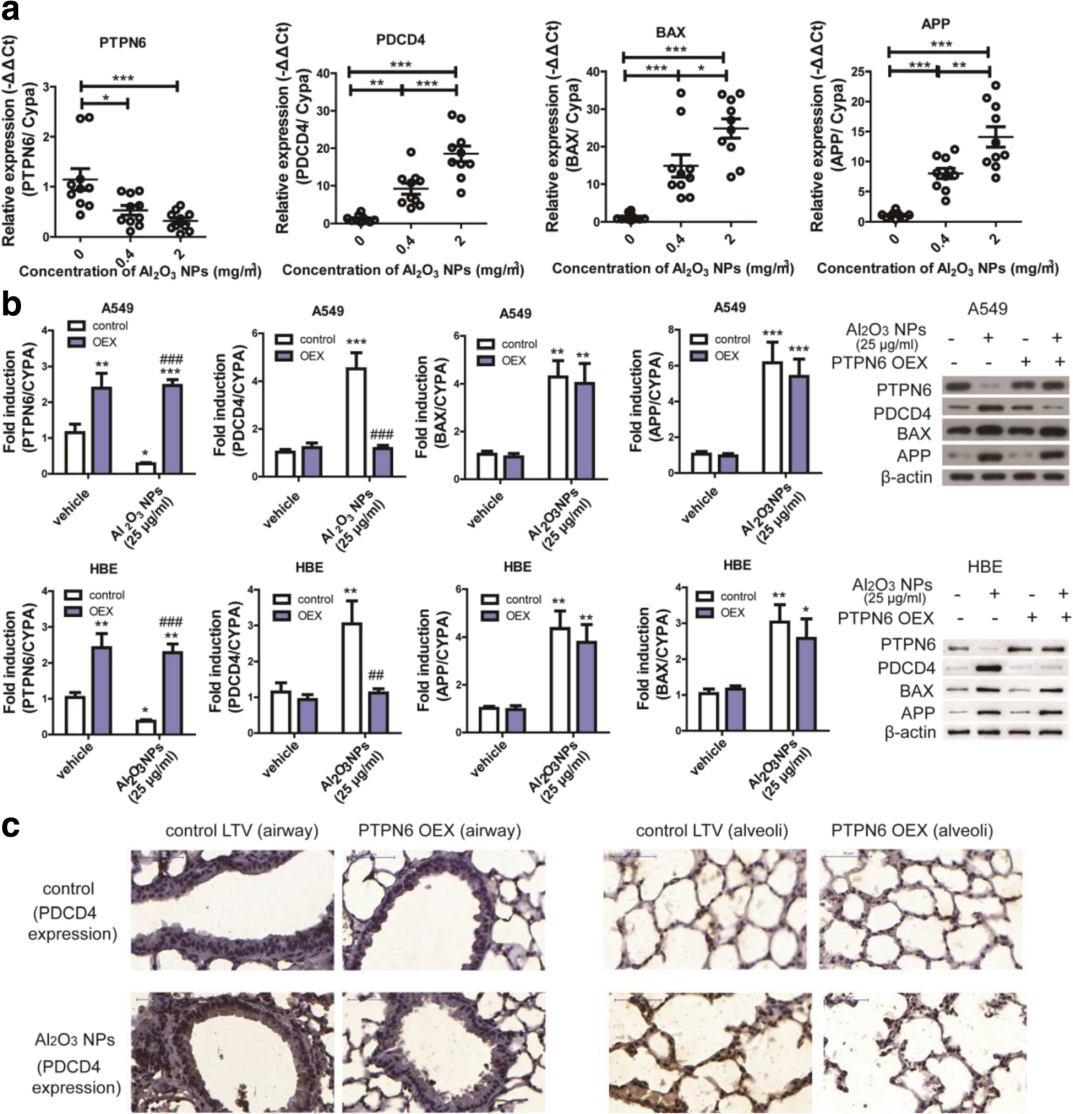
Overexpression of PTPN6 inhibited PDCD4 expression levels both in vitro and in vivo. **a** expression of PTPN6, PDCD4, BAX and APP in mouse lungs exposed to Al_2_O_3_ NPs (*n* = 10, ^*^ *P* < 0.05, ^**^ *P* < 0.01, ^***^ *P* < 0.001). **b** mRNA and protein expression levels of PTPN6, PDCD4, BAX and APP in A549 and HBE cells (*n* = 6, ^*^ *P* < 0.05, compared with vehicle-treated control, ^**^ *P* < 0.01, compared with vehicle-treated control, ^***^ *P* < 0.001, compared with vehicle-treated control, ^##^ *P* < 0.01, compared with vehicle/Al_2_O_3_ NPs-exposed group, ^###^ *P* < 0.001, compared with vehicle/Al_2_O_3_ NPs-exposed group). **c** Representative images of PDCD4 expression in mouse lung tissues. LTV: lentivirus; OEX: overexpression

Our previous study suggested a critical role for PTPN6 in Al_2_O_3_ NP-induced lung injury. Bioinformatics analyses demonstrated that PTPN6 interacted with 3 other genes (PDCD4, BAX, and APP) in the transcriptional factor-gene networks. These 4 genes (PTPN6, PDCD4, BAX and APP) were previously shown to be associated with cell death pathway [14]. Consistent with our observations from Al_2_O_3_ NPs-exposed cells [14], we found that after Al_2_O_3_ NPs exposure lung PTPN6 expression levels were significantly inhibited, and the expression of the other 3 genes (PDCD4, BAX and APP) was significantly increased in a dose-dependent manner (Fig. 3a).

These findings raised the question as to whether the suppression of PTPN6 is only restricted to Al_2_O_3_ NPs-exposure. To address this hypothesis, A549 cells were exposed to two types of nanomaterials, zinc oxide (ZnO) NPs and carbon black (CB) NPs, at concentrations of 0, 25 or 100 μg/mL. Expression levels of BAX were significantly increased in ZnO and CB NPs treated A549 cells, suggesting activation of a universal apoptoic pathway. There were no significant alterations in expression levels of PDCD4, APP or PTPN6 in A549 cells treated by ZnO or CB NPs (Additional file 1: Figure S1b and c). The trends in gene expression levels in ZnO NPs-treated murine lungs were consistent with those noted in ZnO NPs-treated A549 cells (Additional file 1: Figure S1d).

The regulation of PDCD4, BAX and APP by PTPN6 was further validated in two pulmonary cell lines (A549 and HBE). Al_2_O_3_ NPs exposure decreased PTPN6 and increased PDCD4, BAX and APP mRNA as well as protein expression levels. However, overexpression of PTPN6 reduced only the expression of PDCD4 to levels statistically indistinguishable from control (Fig. 3b), indicating inhibition of PDCD4 by PTPN6.

To further validate the regulation of PTPN6 to PDCD4 in vivo, we set up a PTPN6 overexpression mouse model employing intranasal instillation of PTPN6 expression lentivirus. The conditional expression of PTPN6 in murine lungs was significantly increased after intranasal instillation (Additional file 1: Figure S2). Conditional overexpression of PTPN6 in murine lungs inhibited the Al_2_O_3_ NPs-induced increased expression of PDCD4 to levels indistinguishable from control (Fig. 3c, representative images of negative controls for PDCD4 IHC staining are shown in Additional file 1: Figure S3). Thus, the PTPN6/PDCD4 pathway plays a critical role in Al_2_O_3_ NPs-induced experimental COPD in mice.

### Activation of STAT3 mediates Al_2_O_3_ NPs-induced COPD-like lesions

**Fig. 4:**
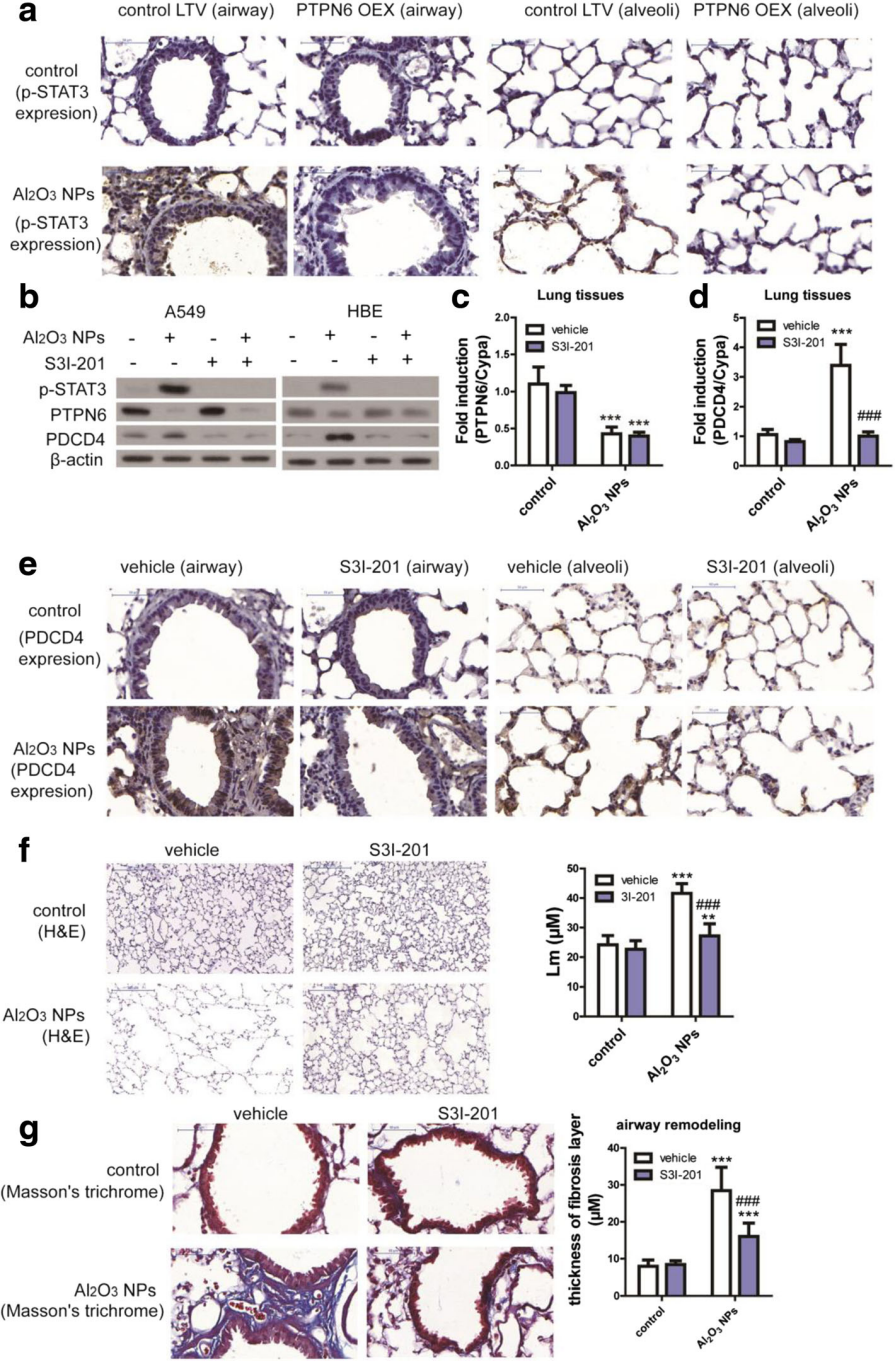
PTPN6 inhibited PDCD4 expression in a STAT3-dependent manner. **a** Representative images of p-STAT3 in mouse lung tissues. **b** The STAT3 inhibitor (S3I-201) inhibited activation of STAT3 and expression of PDCD4 in A549 and HBE cells. **c** The STAT3 inhibitor (S3I-201) did not inhibit expression of PTPN6 in mouse lung tissues (n = 6, ^***^ *P* < 0.001, compared with vehicle control). **d** The STAT3 inhibitor (S3I-201) inhibited expression of PDCD4 in mouse lung tissues (n = 6, ^***^ *P* < 0.001, compared with vehicle control, ^###^ *P* < 0.001, compared with Al_2_O_3_ NPs-treated vehicle mice). **e** Representative images of PDCD4 expression in mouse lung tissues **f** Representative images of emphysema and Lm in mouse lungs (n = 36, ^**^ *P* < 0.01, compared with vehicle control, ^***^ *P* < 0.001, compared with vehicle control, ^###^ *P* < 0.001, compared with Al_2_O_3_ NPs-exposed vehicle mice). **g** Representative images of airway remodeling and thickness of fibrosis layer around airway in mouse lungs (n = 36, ^***^ *P* < 0.001, compared with vehicle control, ^###^ *P* < 0.001, compared with Al_2_O_3_ NPs-exposed vehicle mice)

PTPN6 is a negative regulator to STAT3 signaling [23], and increased PDCD4 expression has been previously shown to be dependent on STAT3 phosphorylation, and subsequently exacerbated inflammation in lung tissues [33]. To address the hypothesis that STAT3 mediated the increased PDCD4 expression induced by PTPN6 inhibition, the expression levels of p-STATs in conditional PTPN6 expression murine lungs were evaluated. As shown in Fig. 4a, expression of p-STAT3 was enhanced in both the airway and alveolar areas following Al_2_O_3_ NPs exposure. Furthermore, PTPN6 overexpression reduced STAT3 phosphorylation. In vitro assays corroborated that an inhibitor of STAT3 activation (S3I-201) effectively inhibited the expression levels of p-STAT3 and PDCD4 following Al_2_O_3_ NPs exposure (Fig. 4b). The expression levels of PTPN6 were not affected by S3I-201 in murine lungs (Fig. 4c). The results from the in vivo studies corroborate the cellular assays, establishing that murine lung PDCD4 expression both at the mRNA and protein levels were significantly reduced by S3I-201 (Fig. 4d and e). Furthermore, we ascertained that inhibition of STAT3 activation partially ameliorated the Al_2_O_3_ NPs-induced emphysema and airway remodeling in murine lungs (Fig. 4f and g). Combined, these results demonstrate that suppression of PTPN6 is associated with increased expression of PDCD4 in murine lung tissues, which was dependent upon the activation of STAT3.

### Over-expression of PTPN6 protects from COPD-like lesions in mice

**Fig. 5:**
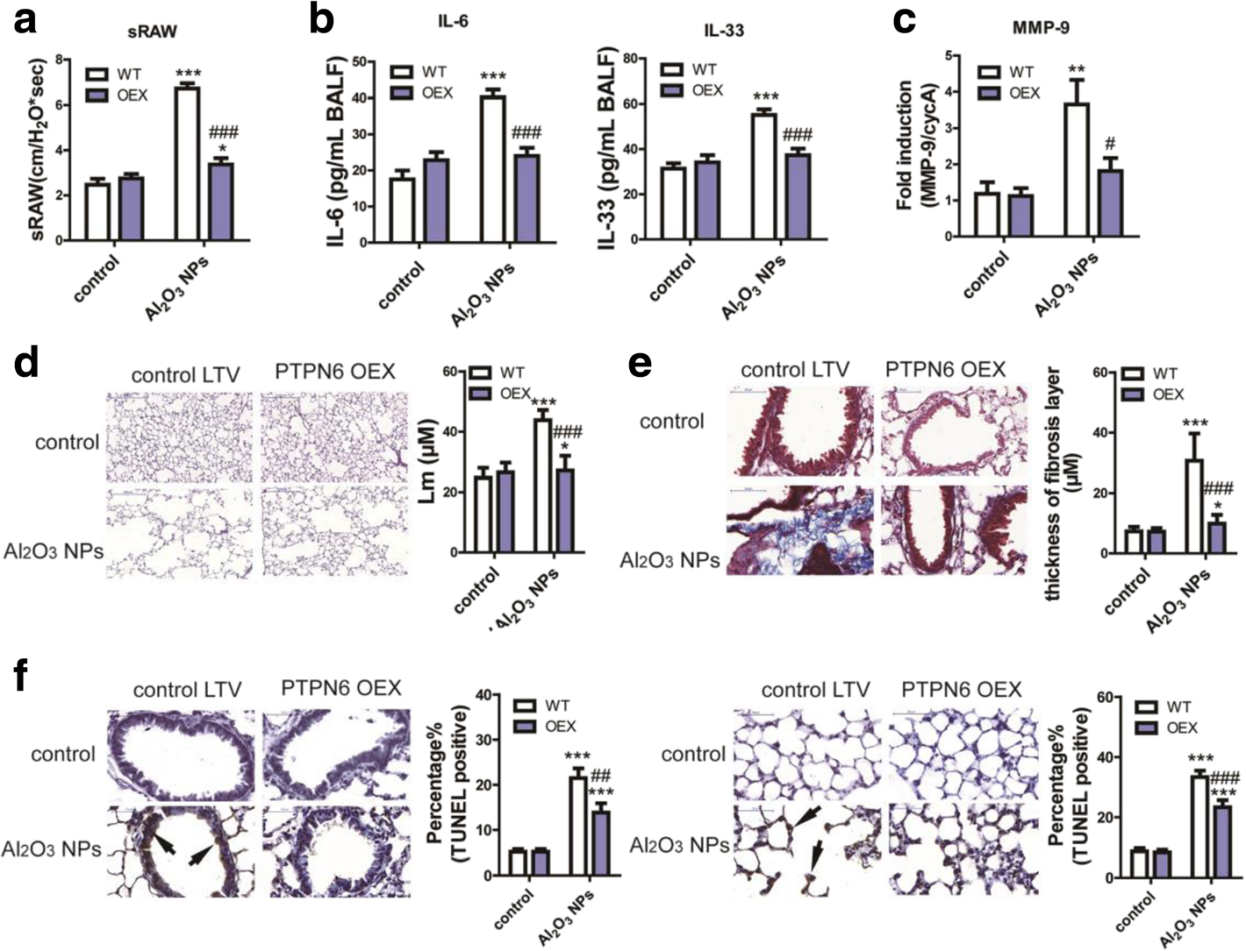
Overexpression of PTPN6 rescued experimental COPD in mouse model. **a** sRAW of conscious mice (n = 10, ^*^ *P* < 0.05, compared with WT control, ^***^ *P* < 0.001, compared with WT control, ^###^ *P* < 0.001, compared with Al_2_O_3_ NPs-exposed WT mice). **b** Levels of inflammatory mediators in BALF (n = 4, ^***^ *P* < 0.001, compared with WT control, ^###^ *P* < 0.001, compared with Al_2_O_3_ NPs-exposed WT mice). **c** Expression levels of MMP-9 in mouse lungs (n = 6, ^**^ *P* < 0.01, compared with WT control, ^#^ *P* < 0.05, compared with Al_2_O_3_ NPs-exposed WT mice). **d** Representative images of emphysema and Lm in mouse lungs (n = 36, ^*^ *P* < 0.05, compared with WT control, ^***^ *P* < 0.001, compared with WT control, ^##^ *P* < 0.01, compared with Al_2_O_3_ NPs-exposed WT mice). **e** Representative images of airway remodeling and thickness of fibrosis layer around airway in mouse lungs (*n* = 36, ^*^ *P* < 0.05, compared with WT control, ^***^ *P* < 0.001, compared with WT control, ^###^ *P* < 0.001, compared with Al_2_O_3_ NPs-exposed WT mice). **f** Representative images of TUNEL staining and percentage of TUNEL^+^ cells in airway epithelia and alveolar epithelia (*n* = 30, ^***^ *P* < 0.001, compared with WT control, ^##^ *P* < 0.01, ^###^ *P* < 0.001, compared with Al_2_O_3_ NPs-exposed WT mice)

As noted above, inhibition of PTPN6 is a critical upstream events in Al_2_O_3_ NPs-induced COPD-like lesions; therefore, the ability of PTPN6 overexpression to rescue COPD-like effects was addressed. Following PTPN6 overexpression, airway responseness (sRAW), IL-6 and IL-33 levels in BALF were significantly reduced (Fig. 5a and b). Mice were also protected from the Al_2_O_3_ NPs-induced inury markers previously shown to be associated with emphysema, with levels of matrix metalloproteinase 9 (MMP9) being indistinguishable from those in mice exposed to filtered room air (FRA) [34]. PTPN6-overexpressing mice were resistant to Al_2_O_3_ NPs-induced changes in the air space enlargement and mean chord length (Fig. 5d), and demonstrated no evidence for small airway remodeling (Fig. 5e). The percentage of apoptotic cells in small airways and alveoli were also accordingly reduced (Fig. 5f). Thus, PTPN6-overexpression protects against emphysema and small airway remodelling, highlighting the functional role of PTPN6 in Al_2_O_3_ NPs-induced lung injury associated with COPD-like effects.

#### Key molecular pathway involved in Al2O3 NPs-induced COPD-like lesions

**Fig. 6:**
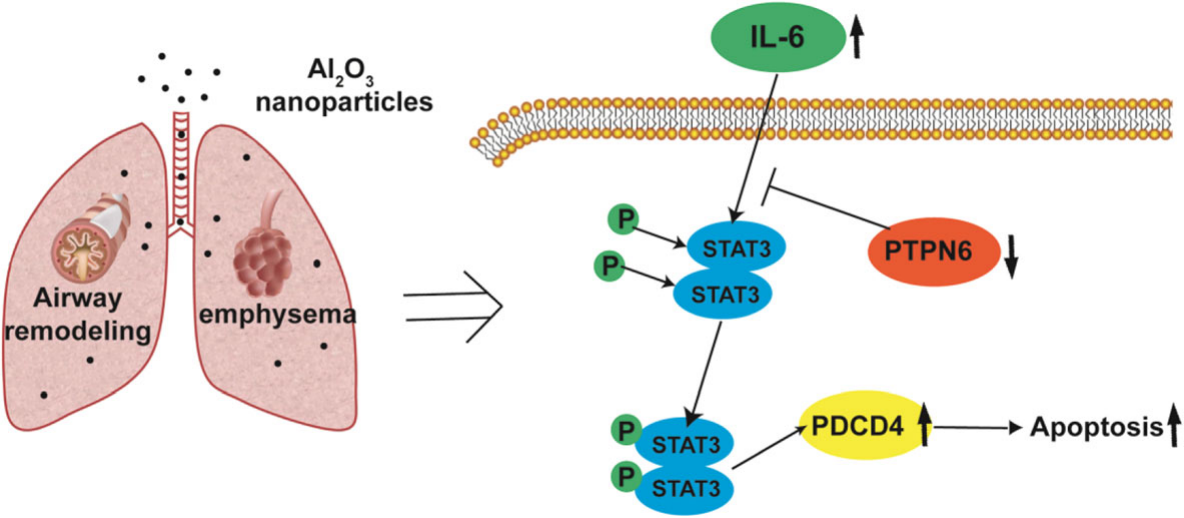
Key molecular pathway involved in Al_2_O_3_ NPs-induced COPD-like lesions

Our results suggest that Al_2_O_3_ NPs exposure induces emphysema and airway remodeling in murine lungs by suppressing PTPN6 expression and enhancing inflammation. In turn, activation of STAT3 increased PDCD4 expression, leading to apoptosis in lung tissue (Fig. 6). Thus, the PTPN6/STAT3/PDCD4 pathway plays a key role in the pathology of COPD-like lesions induced by Al2O3 NPs exposure.

## Discussion

With the wide application of Al_2_O_3_ NPs in industry, agriculture, consumer product and medicine, concerns exist regarding their potential risk to human health and the environment [4]. Al_2_O_3_ NPs release into air may occur during the production and application; therefore, inhalation of NPs is of great concern. Here we establish Al_2_O_3_ NPs dynamic inhalation causes histological alterations, including emphysema and airway remodeling in murine lungs.

In the present study, we noted pulmonary pathology induced by Al_2_O_3_ NPs was more severe than with other Al_2_O_3_-based nanowhiskers [11] or aluminum oxyhydroxides nanoparticles [12, 13]. Increased recruitment of macrophages, but no neutrophilic inflammation or cytotoxicity was observed in murine lungs treated by sub-chronic inhalation of Al_2_O_3_-based nanowhiskers. In this case, it appeared that macrophages were able to control the aerosol load in the pulmonary system in the absence of cytotoxicity [11]. However, the increased recruitment of macrophages did not sufficiently protect lung tissue from Al_2_O_3_ NPs-induced injury, as shown in our studies. In other studies, after 4-week inhalation exposure to Al oxyhydroxides nanoparticles, only the high level (28 mg/m^3^) exposure induced similar inflammatory response in rat lungs [12, 13]. These inconsistences between published reports and our results might be attributable to differences in the composition of the Al nanomaterials, exposure duration and experimental animal species. Pauluhn et al. also suggested that acute pulmonary inflammation after nanoparticle exposure appears to be more closely related to the particle surface area and reactivity [4], corroborating that Al_2_O_3_ NPs, which were used in present study (have higher surface area than nanowhiskers), might cause more severe pulmonary inflammation in comparison to Al_2_O_3_ nanowhiskers.

Chronic obstructive pulmonary disease (COPD) is characterized by poorly reversible airflow obstruction and abnormal inflammatory responses in the lungs. COPD encompasses a variety of pathologic phenotypes, including airway inflammation, emphysema and remodeling of small airways [35, 36]. Cigarette smoke exposure remains the greatest risk factor for COPD; however, at least one-fourth of patients with COPD are non-smokers. In addition, genetic factors and environmental chemicals are strongly suggested to be related to COPD [37]. Artificial NPs, or airborne nano-scaled particles, have been reported to induce chronic pulmonary disorders. Short-term inhalation exposure to copper oxide (CuO) NPs has been shown to cause dose-dependent lung inflammation, as well as histological alterations characterized by emphysema in rats [38]. An established index of COPD, MMP-9, was increased in lung homogenates of newborn mice exposed to TiO_2_ NPs [39]. Even a single dose of intravenous administration of ZnO NPs induced pulmonary emphysema in mice [40]. Here we report, for the first time, that Al_2_O_3_ NPs inhalation induced typical pathological alterations characteristic of COPD, including emphysema and small airway remodeling, corroborating that Al_2_O_3_ NPs represent an environmental risk factor in the etiology of COPD.

The mechanism of COPD remains poorly understood, but involves inflammation and apoptosis [31]. Taking advantage of contemporary bioinformatics approaches, we previously showed that PTPN6 plays a vital role in cellular responses to Al_2_O_3_ NPs exposure [14]. With respect to the up-stream regulator of PTPN6, activation of pro-inflammatory pathway as well as oxidative stress have been reported to suppress the expression of PTPN6 [41, 42]. Suppression of PTPN6 recruits neutrophil to the injury site and increases neutrophil adhesion in vivo*;* however, overwhelming accumulation of neutrophils in the tissue may also cause damage [43]. Consistent with our observations, a recent study reported that STAT3 is activated in lung specimen obtained from patients suffering from severe COPD [19]. Aberrant activation of STAT3 pathway is critical for persistent inflammation in lung tissues, and it has been reported that STAT3 activation is negatively regulated by PTPN6 [44]. Accordingly, we examined whether PTPN6 overexpression could alter responses to Al_2_O_3_ NPs in our experimental COPD model. Overexpression of PTPN6 for the duration of Al_2_O_3_ NPs-exposure protected mice from airway inflammation (by reducing the numbers of total cell, neutrophil and macrophages; as well as the inflammatory mediator levels in BALF). The mice were also protected from emphysema and airway remodeling by PTPN6 overexpression. The protection afforded with a STAT3 inhibitor was similar to that obtained with PTPN6 overexpression (Fig. 4e to g represent the results of STAT3 inhibitor, Fig. 5d to f represent the results of PTPN6 overexpression).

In addition to Al_2_O_3_ NPs, cellulose nanocrystals [45], ZnO NPs [30], iron oxide NPs [46] and nanoparticulate carbon black [47] have been reported to induce emphysema in mouse or rat models; however, the mechanisms involved have yet to be defined. A recent study showed that ~1% of wet lung weight (mg/g) nanoparticle carbon black (average particle size 15 nm) exposure led to enlarged alveolar spaces as well as significantly increased numbers of macrophages, neutrophils and lymphocytes in BALF as compared to vehicle controls. Nanoparticle carbon black induced double-stranded DNA break in phagocytes, therefore activating CD11C^+^ pulmonary antigen presenting cells to secrete pro-T helper 17 cytokines (IL-6 and IL-1β), promoting T helper 17 cell differentiation [47]. Exposures to other metal oxide nanoparticles, such as ZnO and Fe_2_O_3_, have also been associated with COPD-like lesions [30, 46]. Increased expressions of p53, Ras p21 and JNKs are known to be involved in ZnO-induced cellular responses, consistent with samples from COPD patients [30]. Relatively high doses of iron oxide (Fe_2_O_3_) NPs exposure induced pulmonary emphysema, interstitial hyperemia and inflammation, accompanied by enhancement of free radicals and reduction in GSH levels in rat lung tissue [46]. Results presented herein support the hypothesis that suppression of PTPN6 and activation of STAT3 pathway is specifically involved in Al_2_O_3_ NPs-induced COPD-like lesions in mouse model.

Aberrant cell death, such as increased apoptosis, is intensively involved in the pathogenesis of emphysema and small airway remodeling [31, 48]. In Al_2_O_3_ NPs-induced experimental COPD, we found increased PDCD4 expression, a marker of enhanced apoptosis which is associated with macrophage alternative activation and airway remodeling [49]. Under conditions of pulmonary inflammation, PDCD4 is a downstream effector of STAT3 activation [33], corroborating our results. We hypothesize that as a consequence of suppressed expression of PTPN6, STAT3 activation and PDCD4 expression increase in airway and alveolar epithelial cells, leading to apoptosis, inflammation and emphysema in experimental COPD.

Some limitations of our study should be noted. In term of the long-term effects, further time points should be included to explore the clearance of Al_2_O_3_ NPs exposure or reversibility of mice. The in vivo aerosol characterization could be improved by additional details, such as the particle number concentration or size of the aerosol particles. The effects of Al_2_O_3_ NPs coated with different polymers were not evaluated in the present study, which should be considered in the future.

## Conclusions

Taken together, our novel studies provide invaluable new insights into Al_2_O_3_ NPs-specific pulmonary injury. Our results show PTPN6 is down-regulated in response to Al_2_O_3_ NPs-induced experimental COPD. Suppression of PTPN6 may have deleterious effects at the molecular, cellular and tissue levels, leading to initiation of inflammation and apoptosis, ultimately resulting in the development of COPD-like lesions. The molecular cascades of PTPN6/STAT3/PDCD4 play a vital role in Al_2_O_3_ NPs-involved pulmonary disorders. This study raises the possibility of an increased risk of pulmonary disorder upon exposure to Al_2_O_3_ NPs, suggesting the necessity of intensive protection for susceptible populations, especially in occupational settings.

## Methods

### Nanomaterials

Al_2_O_3_ nanoparticles were purchased from Plasmachem GmbH, Germany (purity >99.8%) and stored as nanopowder. The Al_2_O_3_ nanopowder was used in the animal experiments.

The average primary particle size is 40 nm, with a full range of particle size from 5 to 150 nm (manufacturer’s data). The average diameter of Al_2_O_3_ NPs suspended in cell culture medium (DMEM with 10% FBS) with concentration of 25 μg/mL was 77.7 nm following 30 min ultra-sonication, which was tested by zetasizer (nano-zs90, Malvern Instruments, UK) (Additional file 1: Figure S4a). Al_2_O_3_ NPs aggregated in a time-dependent manner and the aggregation size of Al_2_O_3_ NPs with higher concentration was greater than those at lower concentration, as shown in Additional file 1: Figure S4b.

Zinc oxide (ZnO) nanopowders (<100 nm particle size (manufacturer’s data)) were purchased from Sigma-aldrich, USA. The average diameter of zinc oxide (ZnO) nanopowders was 86.37 nm, which were suspended in DMEM medium (with 10% FBS) at a concentration of 25 μg/mL and followed 30 min ultra-sonication (Additional file 1: Figure S4c). Aggregation of ZnO NPs within 24 h is shown in Additional file 1: Figure S4d.

Carbon black (CB) nanopowder was purchased from Plasmachem GmbH, Germany, with an average primary particle size of 13 nm (manufacturer’s data). When suspending in DMEM medium (with 10% FBS) (25 μg/mL) and following 30 min ultra-sonication, the average diameter of CB NPs was 15.1 nm (Additional file 1: Figure S4e). The aggregation of CB NPs in DMEM medium is shown in Additional file 1: Figure S4f.

#### Cell culture and lentivirus transduction

HBE or A549 cells were seeded in 6-well plates at a density of about 1 × 10^6^ cells per well with DMEM medium with 10% FBS.

A549 cells were treated with 0, 25 or 100 μg/ml ZnO NPs or CB NPs for 24 h, mRNA were then collected for qRT-PCR analysis.

PTPN6 overexpression lentivirus was generated by co-transfection with packaging plasmids, pSPAX2 and pMD2G. The overexpression lentivirus harbored a target gene coding sequence, which was tagged with c-Myc. A549 or HBE cells were added with lentivirus (MOI = 30) and treated with Blasticidin S for two weeks to obtain a stable transduction A549 or HBE cells. For experiments, A549 or HBE cells were thawed and allowed to grow for three passages before use. The control A549 or HBE cells and lentivirus stable transduction (LST) cells were then treated with 0 (vehicle) or 25 μg/ml Al_2_O_3_ NPs for 24 h, mRNA and proteins were collected for further analysis.

#### Animals

Male C57BL/6 mice (20–22 g) were purchased from SLRC Laboratory Animal Co., Ltd., China. Animals were treated humanely and all experimental protocols were approved by Committee on Animal Use and Care of Southeast University, China. All the methods in the present study were performed according to approved guidelines. Five mice were housed in each polycarbonate cage with ad libitum access to food and water. Light cycles were set on a 12/12 h light/dark cycle, and room temperature was set at 22.5 °C.

#### Animal experiments

The dynamic inhalation exposure chambers were outfitted with air quality monitoring equipment and a dust aerosol generator (Beijing HuiRongHe Technology Co., Ltd., China). The dry Al_2_O_3_ nanopowder was stored in sample reservoir, and the concentration of dust aerosol was adjusted by the rotation speed of the rotary brush outfitted in the dust aerosol generator (HRH-DAG768, Beijing HuiRongHe Technology, Co. Ltd., China). Exposure was carried out in stainless-steel Hinners-type whole-body inhalation chambers; the treatment groups received Al_2_O_3_ NPs, and the control received high efficiency particulate air (HEPA)-filtered room air (FRA) at the same flow rate as the experimental group. The mass concentrations of Al_2_O_3_ NPs aerosol in the whole-body chamber were measured by a real-time light scattering dust monitor (CEL-712 Microdust Pro, CASELLA CEL Inc., USA), which was placed at the same height as the top of the animal cages. The dust monitor was calibrated using a gravimetric approach by pulling Al_2_O_3_ NPs aerosol through a filter and weighing filter before and after, then dividing mass by the volume of air sampled. The mice were exposed to each chamber for 8 h per day (from 9 a.m. to 5 p.m.) for 7 consecutive days and sacrificed 24 h after the 7-day-treatment. Light cycles were set on at 12/12 h light/dark cycle. The temperature in the chambers was set at 22.5 °C.

The first batch of animal experiments included three groups (with 15 C57BL/6 mice in each group): control exposed to filtered room air (FRA); mice exposed to Al_2_O_3_ NPs with a mean concentration of 0.4 mg/m^3^; or mice exposed to Al_2_O_3_ NPs with a mean concentration of 2 mg/m^3^. The selection of exposure concentration took into account results from a previous inhalation study with Al oxyhydroxides nanoparticles [12], OSHA regulation [6], as well as our previous study [14]. Mice were exposed to Al_2_O_3_ NPs for 7 concecutive days and sacrified 24 h after Al_2_O_3_ NPs exposure. The specific airway resistance (sRAW) was measured after dynamic inhalation on exposure days 3 and 7.

The second batch of mice included two groups (with 10 C57BL/6 mice in each group): control exposed to filtered room air (FRA) and mice exposed to ZnO NPs with a mean concentration of 0.4 mg/m^3^. Mice were exposed to ZnO NPs for 7 concecutive days and sacrified 24 h after ZnO NPs exposure. One piece of lung tissues were fix in 4% PFA and the other lung tissues were stored in liquid nitrogen.

The third batch of mice were divided into four groups (with 10 C57BL/6 mice in each group): control mice treated with FRA and control vector lentivirus; mice treated with FRA and PTPN6 vector lentivirus; mice treated with 0.4 mg/m^3^ Al_2_O_3_ NPs and control vector lentivirus; and mice treated with 0.4 mg/m^3^ Al_2_O_3_ NPs coupled with PTPN6 vector lentivirus. Mice received intranasal treatment with 1 × 10^8^ transducing units (TU)/mouse every three days. Treatment began two days before Al_2_O_3_ NPs exposure. Mice were exposed to Al_2_O_3_ NPs for 7 consecutive days and sacrified 24 h after Al_2_O_3_ NPs exposure.

The fourth batch of mouse included four groups (with ten C57BL/6 mice in each group): control mice treated with FRA and PBS; mice treated with FRA and STAT3 inhibitor S3I-201; mice treated with 0.4 mg/m^3^ Al_2_O_3_ NPs and PBS; and mice treated with 0.4 mg/m^3^ Al_2_O3 NPs coupled with S3I-201. The STAT3 inhibitor, S3I-201 (Millipore Sigma, USA), was dissolved in DMSO (0.05% DMSO) and injected intraperitoneally. Mice received S3I-201 with a dose of 5 mg/kg every three day and began two days before Al_2_O_3_ NPs exposure (totally four times of injection for each mouse). Mice were exposed to Al_2_O_3_ NPs for 7 consecutive days and sacrified 24 h after Al_2_O_3_ NPs exposure. The specific airway resistance (sRAW) was measured after dynamic inhalation on exposure day 7.

#### Mouse airway resistance measurement

The specific airway resistance (sRAW) was evaluated in conscious mice, and was measured using the FinePointe non-invasive airway mechanics (DIS Buxco, USA). Each animal was restrained in a special chamber which allowed for the independent measurement of nasal and thoracic flows. Each mouse was monitored for five consecutive minutes.

#### Aluminum burden

Five mice from each group of the first batch were euthanized under ether anesthesia 1 h after the end of dynamic Al_2_O_3_ NPs exposure on the 7th day. All the mice were decapitated on an iced table. Approximately 0.1 g of lung tissue sample was digested with HNO_3_ in a boiling water bath for 3 h. The aluminum burdens were quantified using an inductively coupled plasma-mass spectrometry (ICP-MS, Agilent 7700, USA). The aluminum burden was calculated as weight/weight of lung tissue (ng/g).

#### BALF isolation, cell counts and ELISA

Four mice from each group were euthanized by ether, the lungs were lavaged with 1.0 ml ice-cold PBS for three times. BALF was centrifuged at 500 g for 5 min. The cell pellet was suspended in 500 μl PBS and leukocytes were counted using a hemocytometer. Eighty μl suspensions were removed for after Wright-Giemsa stain. The percentage of monocytic cells and neutrophils were counted in a total of 300 cells. The levels of IL-6 (R&D system, USA) and IL-33 (R&D system, USA) in BALF were measured with commercial ELISA kits.

#### RNA isolation and quantitative real-time PCR assay

One piece of lung tissue from each mouse was collected and stored in liquid nitrogen for qRT-PCR assay. Total RNA of cells and lung tissues was extracted using a GenElute™ Mammalian Total RNA Miniprep Kit (Sigma, USA) according to the manufacturer’s protocol. The mRNA levels for modulated genes were determined by reverse transcription of total RNA followed by qRT-PCR on a Quant Studio 6 Flex System (Applied Biosystems, Life Technologies, USA) using SYBR PCR Master Mix reagent kits (Takara, Japan) following the manufacturer’s protocol. Primers were designed and provided in Additional file 1: Tables S1. All experiments were independently performed in triplicate. The mRNA levels provided were normalized to cyclophilin A (CYPA).

#### Histopathological analysis of mice lung tissue

One piece of lung tissue from 6 mice in each group were stored in PFA for 24 h at 4 °C, embedded in paraffin, serially sectioned (5 μm) and mounted on silane-covered slides. The sections selected from each mouse were stained after dewaxing with hematoxylin and eosin (H&E) and evaluated under a light microscope (400×) to examine the tissue histology.

The mean linear intercept (L(m)) is quantified to characterize the enlargement of airspaces in emphysema. Six random fields from each section at ×10 magnification under microscopy were qualitifed by the indirect stereological methods [50].

Three of lung sections from each group were stained with Masson’s Trichrome stain and images scanned using the slide scanner Panoramic SCAN (3DHISTECH, Hungary) to obtain a whole slide image. Panoramic Viewer software (3DHISTECH, Hungary) was used to measure the thickness of the sub-epithelial fibrosis layer stained blue by Masson’s Trichrome stain at 12 separate sites around the airway by a blinded experienced pathologist. The mean thickness of the sub-epithelial layer in microns was calculated for airway having a mean internal diameter between 300 and 700 μm [28].

Apoptotic cells in lung tissues were evaluated with terminal-deoxynucleoitidyl transferase mediated nick end labeling (TUNEL) staining by a Roche In Situ Cell Death Detection Kit (Roche, U.S.) according to the suggested protocols. The nuclear stained areas (depicted in dark brown) were identified as TUNEL-positive cell. The proportion of TUNEL-positive cells of alveolar epithelia and bronchial epithelia were estimated by an experienced histologists blinded to treatment conditions. Three to five non-overlapping bronchial tubes and five non-overlapping alveolar areas in each section were counted in high-power fields (HPFS, ×400 magnification) and analyzed. The bronchial tubes or alveolar areas with the maximum number of positive cells were selected for analysis [10].

After dewaxing, IHC staining was performed as previously described [10], and samples were incubated overnight at 4 °C with mouse monoclonal antibodies against PTPN6 (1:100) (ab532559, abcam, USA), p-STAT3 (1:100) (ab76315, abcam, USA) and PDCD4 (ab51495, abcam, USA). Binding to tissue sections was visualized with a biotinylated rabbit anti-mouse IgG antibody (1:400; DAKO) and developed using diaminobenzidine (DAB) as a substrate. For the negative controls, the primary antibodies were omitted.

#### Data analysis

All statistical tests were two-sided and the significance level was set at *P* < 0.05. Significant differences were determined by one-way analysis of variance (ANOVA), followed by Tukey’s multiple comparison tests. Kruskal-Wallis test was used to analyze the mean chord length (Lm) and thickness of fibrosis around small airway. The 2^–ΔΔCt^ method was used to analyze the qRT-PCR results in all experiments. Statistical analysis was performed by SPSS 12.0.

## Additional file

Additional file 1: Supporting information. (PDF 615 kb)

## Abbreviations

Al_2_O_3_: aluminum oxide
BALF: bronchoalveolar lavage fluid
COPD: Chronic obstructive pulmonary disease
CYPA: Cyclophilin A
DAB: Diaminobenzidine
FRA: Filtered room air
H&E: hematoxylin and eosin
HEPA: High efficiency particulate air
HFL1: Human fetal lung fibroblasts
Lm: Mean chord lengths
MMP9: Matrix metalloproteinase 9
NPs: nanoparticles
PAS: Periodic acid-Schiff
PTPN6: Protein tyrosine phosphatase, non-receptor type 6
sRAW: Specific airway resistance
TUNEL: Terminal-deoxynucleoitidyl transferase mediated nick end labeling;
