# Supplementary material for: Suppression of PTPN6 exacerbates aluminum oxide nanoparticle-induced COPD-like lesions in mice through activation of STAT pathway
Source: Part Fibre Toxicol. 2017 Dec 12;14:53. doi: 10.1186/s12989-017-0234-0 (PMC5728016; doi:10.1186/s12989-017-0234-0)

Table S1 Primer sequences for qRT-PCR assays.

Gene symbol	Forward	Reverse
mMmp9	5’CTGGACAGCCAGACACTAAAG3’	5,CTCGCGGCAAGTCTTCAGAG3’
mApp	5’TCCGAGAGGTGTGCTCTGAA3’	5’CCACATCCGCCGTAAAAGAATG3’
mPtpn6	5’GGACTTCTATGACCTGTACGGA3’	5’GCTGCGTGTAATACTCGACCA3’
mPdcd4	5’CCACTGACCCTGACAATTTAAGC3’	5’TTTTCCGCAGTCGTCTTTTGG3’
mBax	5’TGAAGACAGGGGCCTTTTTG3’	5’AATTCGCCGGAGACACTCG3’
mCypa	5’GAGCTGTTTGCAGACAAAGTTC3’	5’CCCTGGCACATGAATCCTGG3’
hBAX	5’CCCGAGAGGTCTTTTTCCGAG3’	5’CCAGCCCATGATGGTTCTGAT3’
hAPP	5’TCTCGTTCCTGACAAGTGCAA3’	5’GCAAGTTGGTACTCTTCTCACTG3’
hPTPN6	5’TGAACTGCTCCGATCCCACTA3’	5’CACGCACAAGAAACGTCCAG3’
hPDCD4	5’ACAGGTGTATGATGTGGAGGA3’	5’TTCTCAAATGCCCTTTCATCCAA3’
hCYPA	5’CCCACCGTGTTCTTCGACATT3’	5’GGACCCGTATGCTTTAGGATGA3’

Figure S1 Gene expression levels in A549 cells.

A: ZnO NPs exposed-lung tissues showed thickened alveolar walls and emphysema.

B: A549 cells exposed to ZnO NPs with concentration of 0, 25, or 100 μg/mL for 24h.

C: A549 cells exposed to CB NPs with concentration of 0, 25, or 100 μg/mL for 24h.

D: Gene expression levels in murine lung tissues exposed to ZnO NPs.

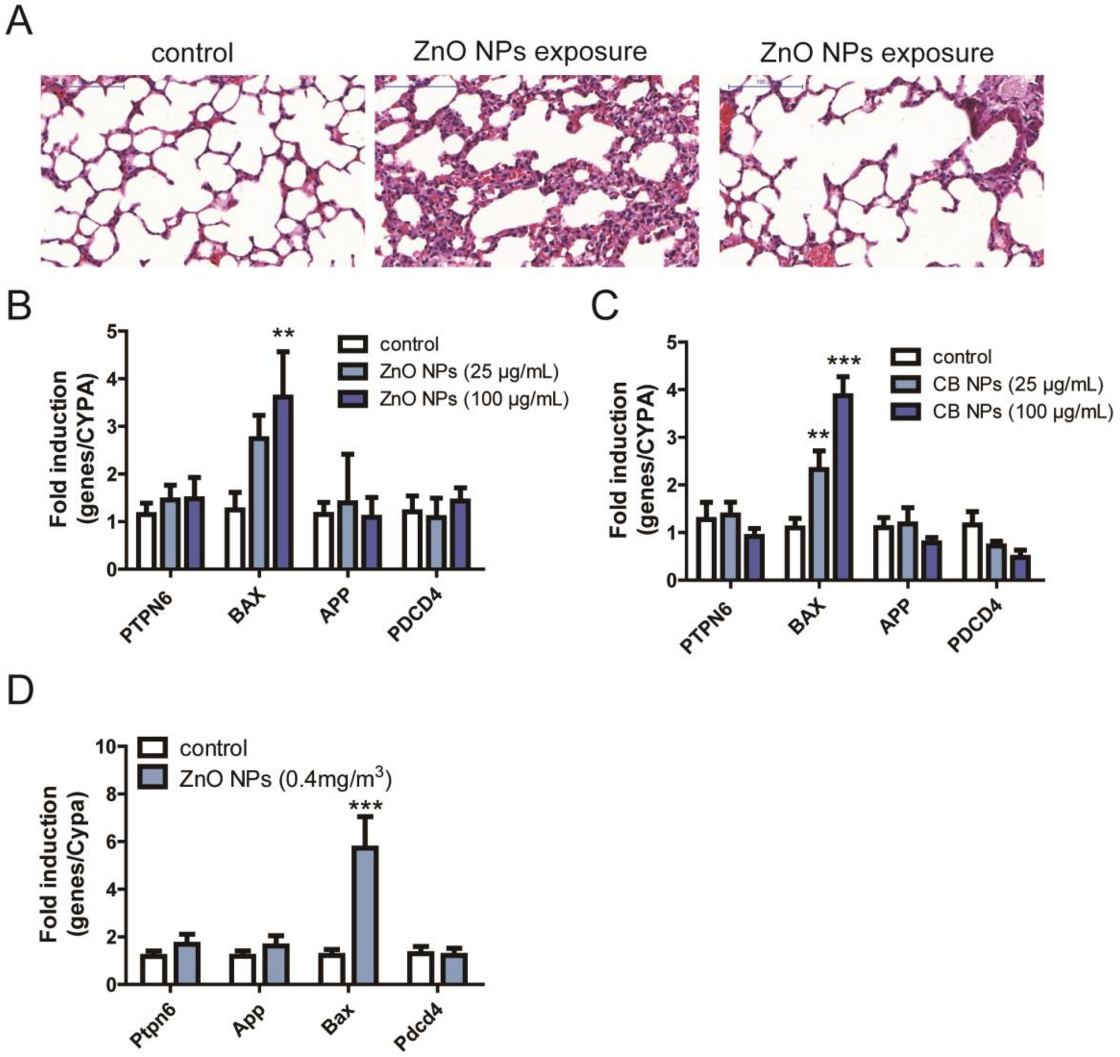

Figure S2 Expression of PTPN6 in mouse lungs following intranasal instillation of PTPN6 expression lentivirus

A: small airway, B: alveolar areas

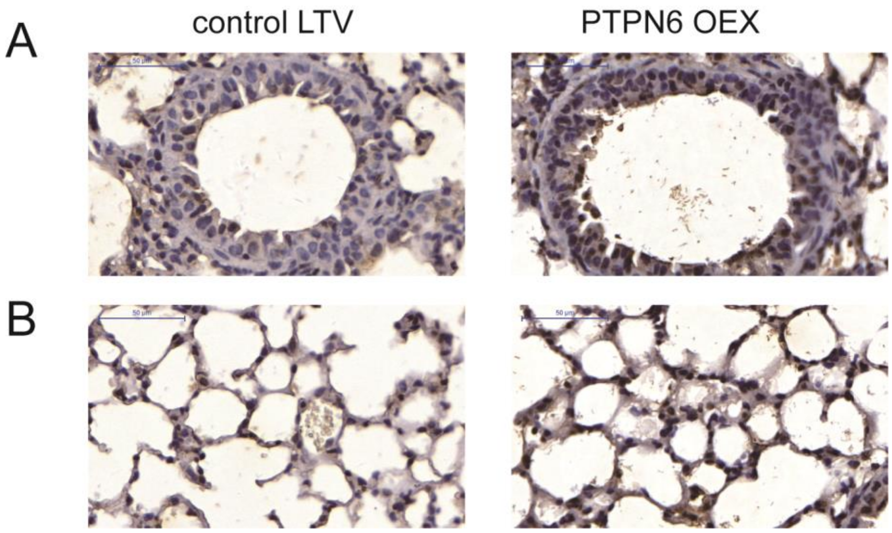

Figure S3 Representative images of negative control for PDCD4 IHC staining.

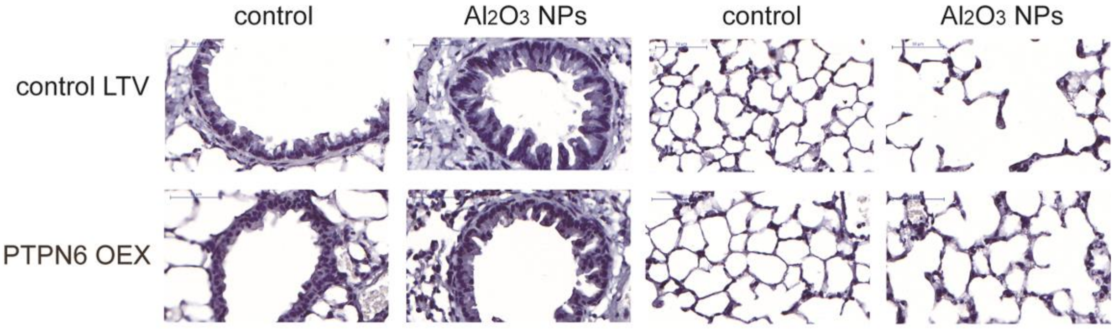

Figure S4 The particle size distribution and aggregation size of nanomaterials suspending in DMEM medium (10% FBS).

A: Particle size distribution of Al_2O_3 NPs at concentration of 25 μg/mL in DMEM medium (10% FBS). B: Aggregation of Al_2O_3 NPs in DMEM medium (10% FBS). C: Particle size distribution of ZnO NPs at concentration of 25 μg/mL in DMEM medium (10% FBS). D: Aggregation of ZnO NPs in DMEM medium (10% FBS). E: Particle size distribution of CB NPs at concentration of 25 μg/mL in DMEM medium (10% FBS). F: Aggregation of CB NPs in DMEM medium (10% FBS).

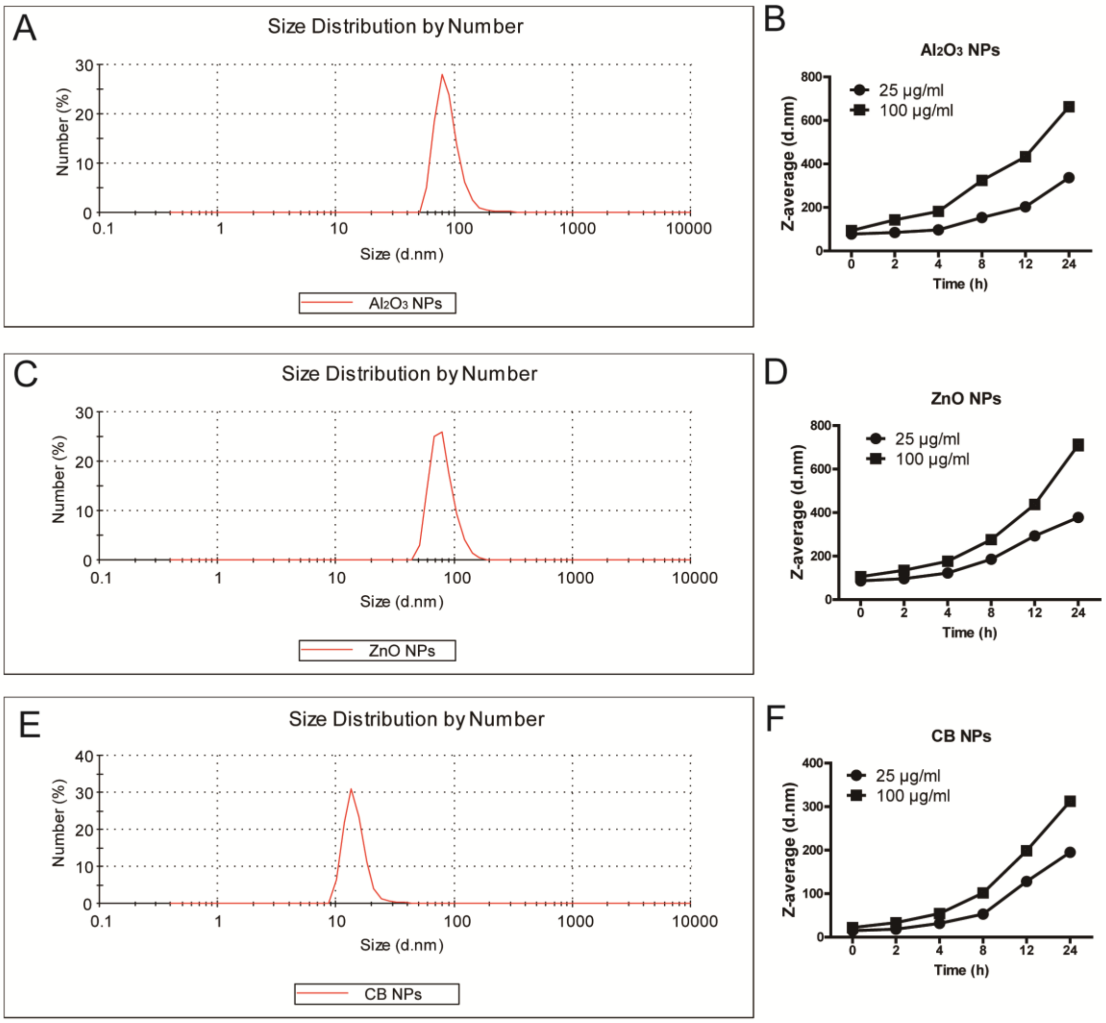

Supplement: Additional file 1: — Supporting information. (PDF 615 kb) [file 12989_2017_234_MOESM1_ESM.pdf]
